# Estimated glomerular filtration rate as one of the main predictors of in-hospital mortality in Egyptian patients with ST elevation myocardial infarction: a two-year retrospective study

**DOI:** 10.1186/s43044-020-00067-z

**Published:** 2020-06-01

**Authors:** Moheb Wadie, Emad Samaan, Mohammed Kamal Nassar, Mostafa Abdelsalam

**Affiliations:** 1 Department of Cardiology, Faculty of Medicine, Mansoura University, Mansoura, Egypt; 2 Mansoura Nephrology and Dialysis Unit, Faculty of Medicine, Mansoura University, Mansoura, Egypt

**Keywords:** Estimated GFR, Shock index, Troponin, CKD-EPI, STEMI

## Abstract

**Background:**

Renal dysfunction is one of the major causes of in-hospital mortality in STEMI patients. In this study, we evaluated the combined predictive value of eGFR by CKD-EPI equation and shock index for in-hospital mortality and other adverse clinical outcomes in Egyptian patients with STEMI.

**Results:**

A total of 450 STEMI patients were divided into 2 groups according to their eGFR with a cutoff value of 60 ml/min/1.73 m^2^ and compared as regards mortality, major bleeding, reinfarction, development of heart failure, stroke, and atrial fibrillation during the period of admission. Univariate analysis was performed to define significant factors that affected mortality; then, significant factors were subjected to a multivariate logistic regression.

Patients with eGFR < 60 ml/min/1.73 m^2^ had higher rates of mortality (*P*  < 0.0005) and atrial fibrillation (*P*  =  .006) during the hospital admission. A multivariate logistic regression model showed the predictors of mortality were factors SI (OR = 28.56, 95% CI 8–101.97, *P* < 0.0001), cardiac troponin (OR = 2.89, 95% CI 1.08–7.77, *P* = 0.03), age (OR = 1.07, 95% CI 1.02–1.2, *P* = 0.002), and eGFR (OR = 0.98, 95% CI 0.96–0.99, *P* = 0.04).

**Conclusions:**

Estimated GFR < 60 ml/min/1.73 m^2^ in STEMI patients is associated with higher rate of mortality. Estimated GFR, age, shock index, and cardiac troponin were the most significant predictors of mortality in STEMI patients

## Background

Coronary artery disease (CAD) is one of the major contributors of morbidity and mortality burden [1]. Within the spectrum of CAD, ST elevation myocardial infarction (STEMI) represents the major risk for adverse events and mortality [2].

Many factors affect the mortality in STEMI patients such as age, heart failure (HF), pre-hospital delay, treatment strategy, diabetes mellitus (DM), anatomy of the affected coronary arteries, and renal function [3]. Constructing risk prediction models from these factors is beneficial for planning management strategies and determining the prognosis [4].

The relationship between renal dysfunction and increased mortality in STEMI patients is well established, and renal function estimation is mandatory to risk stratify STEMI patients [2]. Estimated glomerular filtration ratio (eGFR) by the Modification of Diet in Renal Disease equation has been correlated with prognosis of STEMI patients [5], and eGFR from Chronic Kidney Disease Epidemiology Collaboration (CKD-EPI) equation has been validated as a more simple and accurate marker of renal function [6].

Shock index (SI) is a simple index that means the ratio of heart rate (HR) to systolic blood pressure (SBP). This index has shown a good predictive value for hospital mortality in many critical situations, including trauma [7], pulmonary embolism [8], aortic dissection [9], and STEMI [10].

In the present study, we aimed to assess eGFR by CKD-EPI equation and SI (a simple clinical index) as predictors for in-hospital mortality and other adverse clinical outcomes in Egyptian patients with STEMI from the local STEMI registry and their impact on the initial risk stratification and management in our developing country.

## Methods

This is a retrospective observational study carried out at the Cardiovascular Department. A total of 450 consecutive STEMI patients were recruited between March 2016 and March 2018. All demographic and basic clinical and biochemical characteristics of the STEMI participants were retrospectively analyzed.

Patients who fulfilled the following criteria were included: (a) diagnosis of STEMI according to ACCF/AHA Guideline for the Management of STEMI where 290 patients received thrombolytic therapy,74 treated by primary PCI, 32 had PCI after failed thrombolytic, and 54 patients did not receive thrombolytic therapy or PCI due to late presentation or presence of a contraindication [11] and (b) aged > 18 years and the data were collected from the electronic medical record of specialized medical hospital of cardiovascular department.

### Data collection

All patients were retrospectively analyzed depending on the following: (a) general data, e.g., age, gender, body mass index (BMI), smoking status, duration of hospital admission, and history of other medical disorders; (b) diagnosis on admission, e.g., cardiac condition, cardiac complications (arrhythmia, HF, pulmonary edema), DM, dyslipidemia, and hypertension (HTN); (c) vital data on admission, e.g., SBP, diastolic blood pressure (DBP), and basal HR; and (d) biochemical data on admission to avoid the effect of treatment on the results including serum creatinine, random blood sugar, creatine kinase (CK-MB), qualitative troponin, uric acid, and lipid profile. eGFR was calculated at the time of admission using CKD-EPI 2009 creatinine equation [6]. Physiological predictors as mean arterial pressure (MAP), shock index (SI) = (HR/SBP), modified SI (MSI) = (HR/MAP), and age − SI(age − SI) = (age × SI) were calculated. Killip class was calculated as class I represents those with no signs of HF; class II is for those with rales in the lungs, an S 3 gallop, and elevated jugular venous pressure; class III describes patients with acute pulmonary edema; and class IV describes individuals in cardiogenic shock or hypotension (measured as SBP < 90 mmHg) and evidence of low cardiac output [12].

### Study design

Study participants were divided into two groups based on eGFR at admission: group I (eGFR ≥ 60 ml/min/1.73 m^2^) and group II (eGFR < 60 ml/min/1.73 m^2^).

### Outcome measures

Patients were evaluated for the following outcomes:

- Primary outcome: in-hospital mortality
- Secondary outcomes: occurrence of complications, contrast-induced acute kidney injury (AKI) according to KDIGO definition [13], major bleeding after percutaneous coronary intervention (PCI), in-hospital reinfarction, HF, cerebrovascular accident, and atrial fibrillation (AF)

### Statistical analysis

Data were collected, revised, verified, then analyzed using the Statistical Package of Social Sciences (SPSS) version 21 for Windows (SPSS, Inc., Chicago, IL, USA). Median, mean, and standard deviation (SD) were used for all quantitative values and/or number of cases while percentage for qualitative values. The distribution of tested variables was examined with Kolmogorov-Smirnov test for normality. The significance of differences between continuous variables was determined with independent samples *t* test for parameters with normal distribution and Mann-Whitney test for not normally distributed variables, as appropriate. The chi-square or Fisher exact test was used for comparison between qualitative variables, as appropriate. Univariate analysis was performed to define significant factors that affected mortality; then, 4 significant factors, comprising a mix of demographic, clinical, and laboratory factors, were subjected to a multivariate logistic regression model. The goodness of fit for the model was tested using Hosmer-Lemeshow goodness of fit test (Table 1). Receiver operating characteristic (ROC) curve analysis was done for eGFR to identify the cut points below which mortality is likely. *P* values < 0.05 were considered significant for all statistical analyses in this study.

**Table 1:** Hosmer and Lemeshow test

Step	Chi-square	df	Sig.
1	8.611	8	.376

## Results

The study population were divided into 2 groups according to eGFR on admission: group I with eGFR ≥ 60 ml/min/1.73 m^2^ (354 patients) and group II with eGFR < 60 ml/min/1.73 m^2^ (96 patients).

### Characteristics of the cohort

All the basic and clinical characteristics of the STEMI patients who had undergone PCI are shown in Table 2. Group II patients were significantly older (*P* < 0.0001) and have a higher percentage of female gender, DM, HTN, AF, and history of malignancy or previous hemodialysis (*P* ≤ 0.0001, 0.01, < 0.0001, 0.002, 0.02, and 0.006 respectively) and a lower percentage of smokers (*P* = 0.001), while the other parameters, like dyslipidemia, previous MI, PCI, coronary artery bypass graft surgery, stroke, peripheral vascular disease, congestive HF, and other factors, did not show any significant difference between the two groups.

**Table 2:** Characteristics of the cohort compared according to eGFR levels

Parameter	Group 1 (eGFR ≥ 60 ml/min/1.73 m^2^)	Group 2 (eGFR < 60 ml/min/1.73 m^2^)	*P*
**Number (*n*) (%)**	354 (78.6%)	96 (21.4%)	
**Age (years)**	54.79 ± 10.4	64.94 ± 9.16	< 0.0001**
**Gender**			
**Male**	305 (86.2%)	68 (70.8%)	< 0.0001**
**Female**	49 (13.8%)	28 (29.2%)	
**Smoking**	216 (61%)	40 (41.7%)	0.001*
**Diabetes mellitus**	141 (39.8%)	51 (53.1%)	0.01*
**Hypertension**	122 (34.5%)	55 (57.3%)	< 0.0001**
**BMI (kg/m**^**2**^**)**	29.17 ± 4.99	30.16 ± 5.72	0.12
**AF**	9 (2.5%)	9 (9.4%)	0.002*
**PVD**	1 (0.3%)	2 (2.1%)	0.054
**CHF**	1 (0.3%)	2 (2.1%)	0.054
**Thyroid illness**	2 (0.6%)	2 (2%)	0.13
**Malignancy**	3 (0.8%)	4 (4.2%)	0.02*
**Hemodialysis**	0	2 (2.1%)	0.006*
**Sleep apnea**	1 (0.3%)	0	0.6
**Previous MI**	16 (4.5%)	8 (8.3%)	0.14
**Previous angina**	43 (12.1%)	15 (15.6%)	0.36
**Previous PCI**	12 (3.4%)	4 (4.2%)	0.71
**Previous CABG**	3 (0.8%)	0	0.36
**Previous stroke**	18 (5.1%)	7 (7.3%)	0.4
**Admission duration (days)**	4 (1–13)	4 (1–11)	0.86
**Baseline clinical and laboratory data on admission**			
**HR (beats/minute)**	82.92 ± 19.12	81.66 ± 25.21	0.64
**SBP (mmHg)**	120.36 ± 24.17	117.08 ± 31.31	0.34
**DBP (mmHg)**	76.17 ± 13.96	72.7 ± 18.55	0.04*
**MSI**	0.94 ± 0.32	1 ± 0.46	0.16
**Ejection fraction (%)**	51.34 ± 9.2	49.15 ± 10.41	0.06
**RSWMA**	294 (83.1%)	80 (83.3%)	0.63
**Creatinine (mg/dl)**	0.9 (0.6–1.2)	1.5 (0.7–7.2)	< 0.0001**
**eGFR (ml/min/1.73 m**^**2**^**)**	91.5 ± 17.98	42.88 ± 13.55	< 0.0001**
**RBS (mg/dl)**	177.41 ± 75.68	225.05 ± 109.1	< 0.0001**
**HbA1c (%)**	8.76 ± 2.57	8.95 ± 2.74	0.86
**Uric acid (mg/dl)**	5.85 ± 1.73	7.94 ± 2.05	0.0001**
**Hemoglobin (gm/dl)**	13.99 ± 1.74	13.4 ± 1.98	0.005*
Neutrophil*10^**3**^	8.5 (0.62–26.5)	8.9 (0.9–26)	0.62
Lymphocyte*10^**3**^	2.1 (0.39–19.4)	1.7 (0.29–17.23)	0.17
**Nutrophil/lymphocyte ratio (NLR)**	2.8 (0.52–17.2)	2.67 (0.6–4.74)	0.55
Platelets*10^**3**^	241 (66–574)	216 (32–752)	0.08
**CK-MB (IU/L)**	60 (12–1150)	80.5 (1–876)	0.03*
**Positive troponin (qualitative test)**	183 (51.7%)	67 (69.8%)	0.001*
**Total cholesterol (mg/dl)**	203.41 ± 50.86	185.43 ± 41.4	0.004*
**LDL-cholestrol (mg/dl)**	130.7 ± 37.51	112.43 ± 34.11	0.008*
**HDL-cholesterol (mg/dl)**	43.92 ± 16.11	46 ± 17.3	0.8
**Triglycerides (mg/dl)**	144 (35–711)	134.5 (60–792)	0.15

Data expressed as mean ± SD, median (min-max), or number (%)

*BMI* body mass index, *AF* atrial fibrillation, *PVD* peripheral vascular disease, *CHF* congestive heart failure, *MI* myocardial infarction, *PCI* percutaneous coronary intervention, *CABG* coronary artery bypass grafting, *HR* heart rate, *SBP* systolic blood pressure, *DBP* diastolic blood pressure, *MSI* modified shock index, *RSMWA* resting segmental wall motion abnormalities, *eGFR* estimated glomerular filtration rate, *RBS* random blood sugar, *LDL* low-density lipoprotein, *HDL* high-density lipoprotein

*P < 0.05

**P < 0.0001

Group II patients had significantly lower DBP, eGFR, hemoglobin (HB), total cholesterol, and low-density lipoprotein cholesterol (*P* = 0.04, < 0.0001, 0.005, 0.004, and 0.008 respectively) and a significantly higher serum creatinine, random blood sugar, uric acid, CK-MB, and percentage with positive troponin on admission (*P* ≤ 0.0001, < 0.0001, < 0.0001, 0.03, and 0.001 respectively). Other parameters like HR, SBP, ejection fraction, and others did not have any statistically significant difference between both groups **(**Table 2**)**.

Regarding the primary outcome of this study, mortality was significantly higher in group II (*P* < 0.0001) (Table 3). The highest mortality rate was observed in patients who did not receive any reperfusion therapy [*n* = 12 (22.2%)] with no significant difference between other types of reperfusion therapy (Table 4). Regarding the secondary outcomes, none of our patients developed AKI after contrast administration. In-hospital HF and AF were significantly higher in group II (*P* = 0.02 and 0.006 respectively), while major bleeding, in-hospital reinfarction, and cerebrovascular accident did not differ between both groups.

**Table 3:** Frequencies of different outcomes compared as regards eGFR levels

Parameter	Group 1 (GFR ≥ 60 ml/min) 354 (78.6%)	Group 2 (GFR < 60 ml/min) 96 (21.4%)	*P*
**Mortality*n*(%)**	**17 (4.8%)**	**19 (19.8%)**	< 0.0001**
**Major bleeding**	**8 (2.3%)**	**5 (5.2%)**	**0.16**
**Reinfarction (in-hospital)**	**2 (0.6%)**	**1 (1%)**	**0.5**
**Heart failure (in-hospital)**	**21 (5.9%)**	**12 (12.5%)**	0.02*
**Stroke (in-hospital)**	**4 (1.1%)**	**3 (3.1%)**	**0.17**
**AF (in-hospital)**	**9 (2.5%)**	**9 (9.4%)**	0.006*

Data expressed as number (%)

*AF* atrial fibrillation

*P < 0.05

**P < 0.0001

**Table 4:** Effect of type of reperfusion therapy on mortality

Reperfusion therapy	No. (%)	Mortality No. (%)	*P*
**No reperfusion**	**54 (12)**	**12 (22.2)**	0.001*
**Thrombolytic therapy**	**290 (64.4)**	**18 (6.2)**	
**Primary PCI**	**74 (16.4)**	**5 (6.7)**	
**Rescue PCI**	**32 (7.1)**	**1 (3.1)**	

Data expressed as number (%)

*PCI* percutaneous coronary intervention

*P < 0.05

Significant predictors that affected mortality on a univariate analysis of 16 clinical, laboratory, and physiological parameters were age (*P* < 0.0001), sex (*P* = 0.002), HB (*P* < 0.0005), cardiac troponin (*P* = 0.006), CK-MB (*P* = 0.04), eGFR (*P* < 0.1), MAP, SI, MSI, AGE-SI, and Killip (*P* < 0.0001) (Table 5).

**Table 5:** Univariate logistic regression in prediction of mortality in the studied group

Variable	Coefficient (*β*)	*P*	OR (95% CI)
**Clinical predictors**			
**Age (years)**	**0.074**	< 0.0001**	**1.077 (1.04–1.114)**
**Gender**	**1.129**	0.002*	**3.091 (1.489–6.417)**
**Diabetes mellitus**	**0.443**	**0.204**	**1.557 (0.787–3.082)**
**Hypertension**	**− 0.420**	**0.264**	**0.657 (0.315–1.372)**
**Laboratory predictors**			
**Hemoglobin (gm/dl)**	**− 0.400**	< 0.0001**	**0.670 (0.549–0.819)**
**NL-ratio**	**− 3.982**	**0.424**	**0.019 (0.00–323.363)**
**Cardiac troponin (positive)**	**1.277**	0.006*	**3.585 (1.44–8.922)**
**CK-MB (IU/ml)**	**0.002**	0.041*	**1.002 (1–1.003)**
**eGFR (ml/min/1.73 m**^**2**^**)**	**− 0.034**	< 0.0001**	**0.967 (0.954–0.98)**
**Physiological predictors**			
**SI**	**2.782**	< 0.0001**	**16.151 (5.801–44.962)**
**MSI**	**2.129**	< 0.0001**	**8.411 (3.935–17.976)**
**Killip score**	**1.323**	< 0.0001**	**3.755 (2.753–5.121)**
**Age-SI**	**.056**	< 0.0001**	**1.057 (1.038–1.077)**
**MAP**	**− .057**	< 0.0001**	**0.945 (0.925–0.966)**

*OR* odds ratio, *CI* confidence interval, *NL-ratio* neutrophil lymphocyte ratio, *eGFR* estimated glomerular filtration rate, *SI* shock index, *MSI* modified shock index, *Age-Si* age shock index, MAP mean arterial pressure

*P < 0.05

**P < 0.0001

**Fig. 1:**
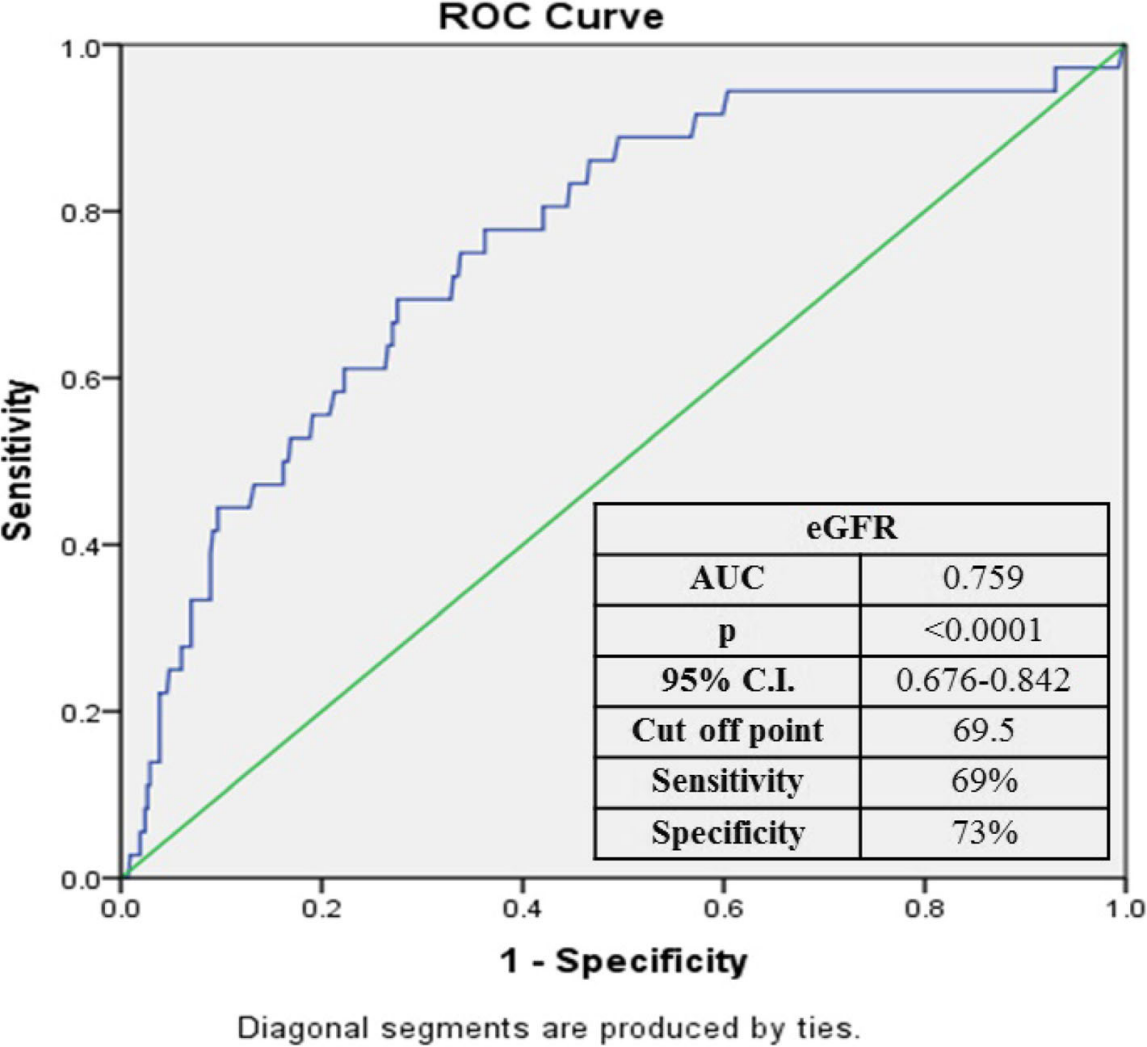
ROC curve for eGFR as a predictor of mortality. eGFR, estimated glomerular filtration rate; CI, confidence interval

Multivariate logistic regression model defining significant predictors for mortality was highly significant (*P* < 0.0001) (Table 6). The significant factors were SI (*P* < 0.0001, OR = 28.56), cardiac troponin (*P* = 0.03, OR = 2.89), age (*P* = 0.002, OR = 1.07), and eGFR (*P* = 0.04, OR = 0.98) (Table 6). ROC curve analysis was done for eGFR with a value of 69.5 ml/min/1.73 m^2^ or less identifies the probability of mortality with 69% sensitivity and 73% specificity (Fig. 1).

**Table 6:** Multivariate logistic regression model in prediction of mortality in the studied group

Variable	Coefficient (*β*)	*P*	OR (95% CI)
**SI**	**3.352**	< 0.0001**	**28.562 (8–101.979)**
**Cardiac troponin (positive)**	**1.064**	0.035*	**2.898 (1.081–7.772)**
**Age (years)**	**.069**	0.002*	**1.072 (1.026–1.2)**
**eGFR (ml/min/1.73 m**^**2**^**)**	**− .018**	0.041*	**0.982 (0.965–0.999)**
**Constant**	**− 8.941**	< 0.0001**	**0.000**

*SI* shoch index, *eGFR* estimated glomerular filtration rate, *OR* odds ratio, *CI* confidence interval

*P < 0.05

**P < 0.000

## Discussion

STEMI has high morbidity and mortality. Risk stratification is of extreme importance for better patient outcome [14]. Our goal was to test some of the previously reported predictors of mortality in STEMI patients in an Egyptian cohort. We specified 16 risk factors according to the data available in our archive. They were divided into clinical, biochemical, and physiological predictors. Univariate analysis was performed to define significant factors that affected mortality. We then selected 4 factors with the highest coefficients and odds ratios to construct a multivariate prediction model. The selection of only four factors was based on the standard statistical equation for building a well-fitted multivariate prediction model respecting the tested variables to sample size ratio [15]. We have paid special attention to eGFR calculated by CKD-EPI equation as an independent predictor of mortality and other adverse outcomes.

### Clinical predictors of mortality

#### Effects of age and gender on the in-hospital prognosis of STEMI patients

The age was an independent risk factor of mortality in the present study, which is similar to previous recently published reports [16, 17]. Logically, aging is associated with chronic comorbidities and pathological changes in some organs, which affect the patient outcome and response to tissue injury and treatment, either directly or indirectly. Recent results of a systematic review and meta-analysis of 24 studies (430,914 STEMI patients) showed that the in-hospital mortality of male patients was significantly lower than that of females [18]. In consistence with these observations, we reported the same worse prognosis for females than males. In our cohort, the STEMI females were significantly older than the STEMI males. Also, the rates of having other diseases such as DM and HTN were higher in females. Previous studies showed that DM [19], HTN [20], and dyslipidemia were significant risk factors of mortality in STEMI patients. We did not find, any of them, having a significant impact on in-hospital mortality. Based on our analysis of the clinical predictors, we chose the age factor to share in the multivariate prognostic model construction for prediction of mortality in our cohort.

#### Effects of eGFR by CKD-EPI equation on the in-hospital prognosis of STEMI patients

The renal dysfunction in STEMI patients is a well-known predictor for the in-hospital and long-term mortality [21]. Also, the level of serum creatinine is one of the prognostic predictors after treatment [11, 22]. This is, in addition to, the interest of many studies to question the role of the various known equations that estimate the GFR to predict the patient outcomes [23]. The currently recommended equation for the estimation of GFR is CKD-EPI [6]; our study showed that reduced eGFR using this equation significantly affected the in-hospital prognosis of STEMI and eGFR below 60 ml/min was associated with about 4-fold increase in the mortality rate compared with those with eGFR above 60 ml/min. The rates of in-hospital incident HF and AF are also significantly higher in those with eGFR below 60 ml/min. This could be explained by the fact that lower eGFR levels are associated with advanced age, HTN, and DM in addition to the impact of nephropathy itself due to extensive atherosclerosis, calcifications, multiple organ affection, and limitation of medication such as ACE or ARBs. Therefore, eGFR should be considered as important predictors of prognosis, and risk stratifications should be conducted based on the functional status of the kidneys to reduce mortality and improve the outcome in STEMI patients.

#### Effects of admission vital signs and physiological predictors on the in-hospital prognosis of STEMI patients

Vital signs have received considerable research interest in the epidemiological studies concerned with STEMI risk prediction because of their importance in expressing the physiological and pathological cardiac changes. Tachycardia reflects the sympathetic overactivity, high myocardial oxygen consumption, and myocardial work in patients with STEMI [24, 25]. The blood pressure reflects the extent of myocardial damage and subsequently predicts the mortality [26].

The combination of more than one factor is always better than relying on a single one, and this motivated many researchers to test multiple indices with different components of vital signs to predict the risk magnitude among STEMI patients. Of those, SI [27] and MSI [28, 29] were the most proven to have a significant relation to mortality and adverse outcomes in STEMI with higher prediction power of MSI over the SI [30]. High Killip class was also described as a predictor of mortality in STEMI patients [31]. In the present study, we found that SI, MSI, MAP, SI-AGE, and Killip score were all significantly related to mortality with the highest coefficient and odds to the SI. Based on these results, SI was chosen for the multivariate prediction model.

#### Effects of admission cardiac enzymes and hemoglobin on the in-hospital prognosis of STEMI patients

The value of cardiac enzymes represents the infarction extent and size and is associated with the magnitude of myocardial necrosis and in-hospital mortality in STEMI patients [32]. Our study reported that either cardiac troponin positivity or the increase in CK-MB levels was associated with increased mortality risk with higher odds for troponin positivity. This is in contrast to a published report that showed higher predictability and prognostic significance for CK-MB levels [15].

In patients of acute STEMI, low baseline HB behaved independently as a risk factor for increased 30-day event rates [33]. In line with this report, we also found a positive correlation between the lower HB levels and mortality in STEMI patients.

Multivariate model was constructed from combination of clinical, laboratory, and physiological predictors of the highest coefficients and odds. We found that the most significant predictors of in-hospital mortality for STEMI in our setting were SI, cardiac troponin positivity, age, and eGFR. The model has the advantage of ease of application as it is based on simple clinical signs and routine laboratory assessment.

There were few limitations of our study. First, our study was a single-center study. Second, we used the qualitative test of cardiac troponin due to limited resources. Third, it had short-term follow-up till the patients’ discharge only. Although the results were significant in such period, short- and long-term follow-up analysis is required, which is undergoing and will be stated.

## Conclusion

In conclusion, eGFR, age, SI, and cardiac troponin are valid to construct a prediction model for mortality in STEMI patients. However, more studies including higher number of patients, from different centers, and longer follow-up are required.

## Data Availability

All data generated or analyzed during this study are available from the corresponding author on reasonable request

## Abbreviations

AF: Atrial fibrillation
BMI: Body mass index
CAD: Coronary artery disease
CKD-EPI: Chronic Kidney Disease Epidemiology Collaboration
CK-MB: Creatine kinase MB fraction
DBP: Diastolic blood pressure
DM: Diabetes mellitus
eGFR: Estimated glomerular filtration ratio
HB: Hemoglobin
HF: Heart failure
HR: Heart rate
HTN: Hypertension
MAP: Mean arterial pressure
MSI: Modified shock index
PCI: Percutaneous coronary intervention
SBP: Systolic blood pressure
SI: Shock index
STEMI: ST elevation anterior myocardial infarction
